# Aortic remodelling induced by obstructive apneas is normalized with mesenchymal stem cells infusion

**DOI:** 10.1038/s41598-019-47813-1

**Published:** 2019-08-07

**Authors:** Cira Rubies, Ana-Paula Dantas, Montserrat Batlle, Marta Torres, Ramon Farre, Gemma Sangüesa, Josep M. Montserrat, Lluis Mont, Isaac Almendros, Eduard Guasch

**Affiliations:** 1 Institut d’Investigacions Biomèdiques August Pi i Sunyer (IDIBAPS), Barcelona, Spain; 2 Centro de Investigación Biomédica en Red de Enfermedades Cardiovasculares (CIBERCV), Madrid, Spain; 3 Unitat del Son. Servei Pneumologia, Hospital Clínic. Universitat de Barcelona, Barcelona, Spain; 4 Unitat de Biofísica i Bioenginyeria, Facultat de Medicina i Ciències de la Salut, Universitat de Barcelona, Barcelona, Spain; 5 Centro de Investigación Biomédica en Red de Enfermedades Respiratorias (CIBERES), Madrid, Spain; 6 Institut Clínic Cardiovascular, Hospital Clínic, Universitat de Barcelona, Barcelona, Spain

**Keywords:** Vascular diseases, Respiratory tract diseases, Experimental models of disease

## Abstract

Obstructive sleep apnea syndrome (OSA) promotes aortic dilatation, increased stiffness and accelerated atherosclerosis, but the mechanisms of vascular remodelling are not known. We aimed to assess vascular remodelling, its mechanisms, and the effect of mesenchymal stem cells (MSC) infusions in a clinically relevant rat model of chronic OSA involving recurrent airway obstructions leading thoracic pressure swings and intermittent hypoxia/hypercapnia (OSA-rats). Another group of rats were placed in the same setup without air obstructions (Sham-rats) and were considered controls. Our study demonstrates that chronic, non-invasive repetitive airway obstructions mimicking OSA promote remarkable structural changes of the descending thoracic aorta such as eccentric aortic hypertrophy due to an increased wall thickness and lumen diameter, an increase in the number of elastin fibers which, in contrast, get ruptured, but no changes in tunica media fibrosis. As putative molecular mechanisms of the OSA-induced vascular changes we identified an increase in reactive oxygen species and renin-angiotensin system markers and an imbalance in oxide nitric synthesis. Our results also indicate that MSC infusion blunts the OSA-related vascular changes, most probably due to their anti-inflammatory properties.

## Introduction

Obstructive sleep apnea syndrome (OSA) is a common sleep-related breathing disorder characterized by recurrent episodes of partial or complete obstruction of the upper airway. Numerous studies have identified severe OSA as a risk factor for hypertension, heart failure, stroke, and mortality^1^. Moreover, growing evidence suggests that untreated OSA accelerates atherosclerosis^2^, which may mediate the increased cardiovascular disease burden in OSA patients. Several mechanisms linking OSA with vascular complications have been postulated, including intermittent hypoxia (IH) and hypercapnia, intrathoracic swings, repeated arousals, and sympathetic system activation, all them potentially acting via inflammation, endothelial dysfunction, and oxidative stress^3^.

In these patients, comorbidities such as obesity, diabetes and hypertension often coexist with, or are induced by, OSA, and hamper establishing an independent association between OSA and the progression of atherosclerosis. The use of animal models overcomes the confounding effects of comorbidities and enables the exploration of the pathophysiological consequences of OSA. The most widely used animal model of sleep apnea selectively recapitulates IH^4–6^. Although IH is an important characteristic of OSA, this model is limited by the absence of recurrent upper airway obstructions and consequent deep intrathoracic pressure swings. Some alternative models need of an invasive approach to induce airway obstruction, limiting the exploration of the long-term consequences of OSA^7,8^. However, our group has developed a setup that non-invasively reproduces recurrent airway obstructions^9^, resulting in increased breathing efforts, oxygen desaturations and intermittent hypercapnia mimicking severe OSA (apnea-hypopnea-index [AHI] > 30 per hour).

Eventually, a systemic pro-inflammatory environment^10^ that locally extends to the cardiovascular system^11,12^ seems to be a major contributor to OSA-related deleterious cardiovascular outcomes. In this setting, bone marrow mesenchymal stem cells (MSC) appear a promising therapeutic approach because of their anti-inflammatory^13^ and anti-fibrotic^12,14^ effects. Of note, MSC are mobilized into circulating blood early in the course of OSA^15^. The putative therapeutic effect of MSC on atrial fibrosis and systemic inflammation has been previously demonstrated in our OSA rat model^12,13^.

Our objectives in the present study were (a) to assess vascular remodelling after long-term repetitive obstructive apneas in a clinically relevant rat model, (b) to study its mechanisms, and (c) to test the effects of MSC infusion in the progression of OSA-induced deleterious vascular remodelling.

## Results

### Morphological vascular remodelling induced by OSA

**Figure 1:**
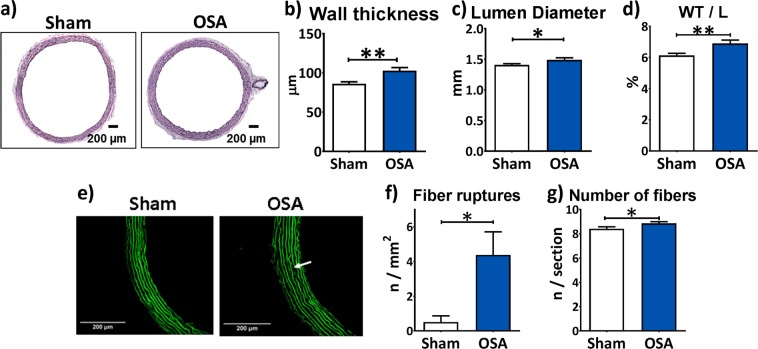
Aortic morphological remodelling induced by OSA. Representative haematoxylin-eosin stained aortic sections from Sham and OSA rats (**a**). Column graph of aortic wall thickness (µm, **b**), lumen diameter (mm, **c**), wall thickness to lumen ratio (%, **d**) from Sham (n = 11, white columns) and OSA (n = 12, blue columns) rats. Representative elastin auto fluorescence images from aorta wall from Sham and OSA rats (**e**). White arrow in the picture from an OSA rat shows an elastic fiber rupture. Number of fiber ruptures indexed per area (Fiber ruptures/mm^2^, **f**) and number of elastic fibers relative to aorta section (**g**) in Sham (n = 12, white columns) and OSA (n = 16, blue columns) rats. Data are expressed as mean ± SEM, *p < 0.05, **p < 0.01.

Recurrent apneas prompted evident changes in descending aorta morphology and structure. A representative microphotograph is shown in Fig. 1a. The aortic wall thickness (WT, Fig. 1b) and lumen diameter (Fig. 1c) were significantly increased in OSA compared with Sham rats. Overall, WT thickening prevailed, as denoted by a higher WT to lumen ratio (WT/L, Fig. 1d) in OSA rats.

We subsequently studied elastic fiber organization in the descending thoracic aorta (Fig. 1e). Our analyses demonstrated a significant increase of elastic fiber ruptures in OSA rats compared to Sham rats (Fig. 1f). OSA rats had more elastic fibers in the aorta than Sham rats (Fig. 1g).

**Figure 2:**
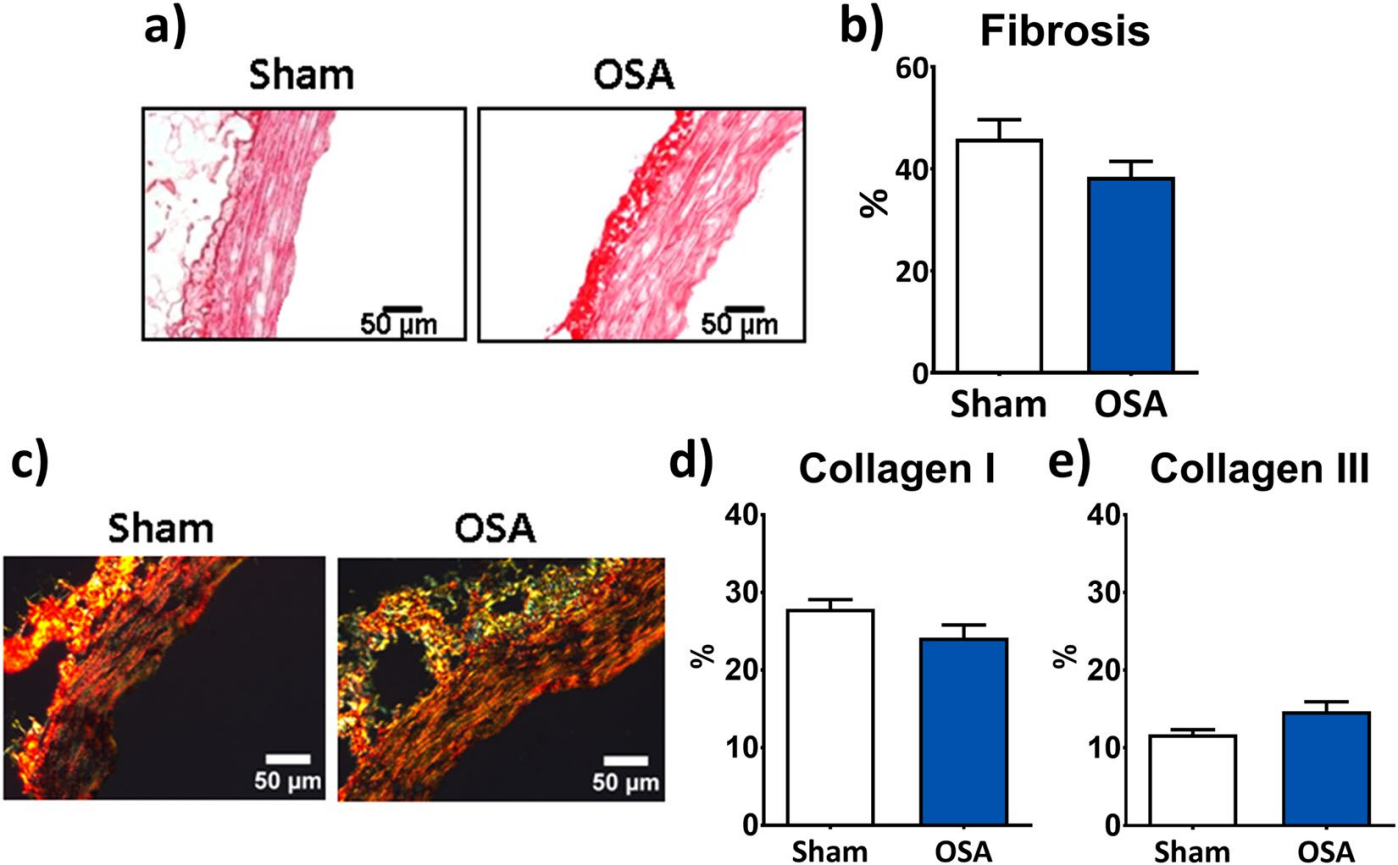
Aortic collagen content. Representative picrosirius-stained aortic OCT sections of Sham and OSA rats (**a**) and column graph of the quantification of percentage of collagen deposition (**b**) of Sham (n = 11, white columns) and OSA rats (n = 15, blue columns). Representative picrosirius-stained aortic OCT sections obtained under polarized light are shown in (**c**). Column graphs of collagen I (**d**) and collagen III (**e**) quantification of Sham (n = 7, white columns) and OSA rats (n = 8, blue columns).Data are expressed as mean ± SEM.

Tunica media fibrosis in the aorta was assessed by means of Picrosirius red staining. Quantification of collagen deposition did not show differences between Sham and OSA groups (Fig. 2a,b). Collagen subtype analysis with polarized light (Fig. 2c) demonstrated similar deposition of each collagen I and III (Fig. 2d,e, respectively) between groups.

**Figure 3:**
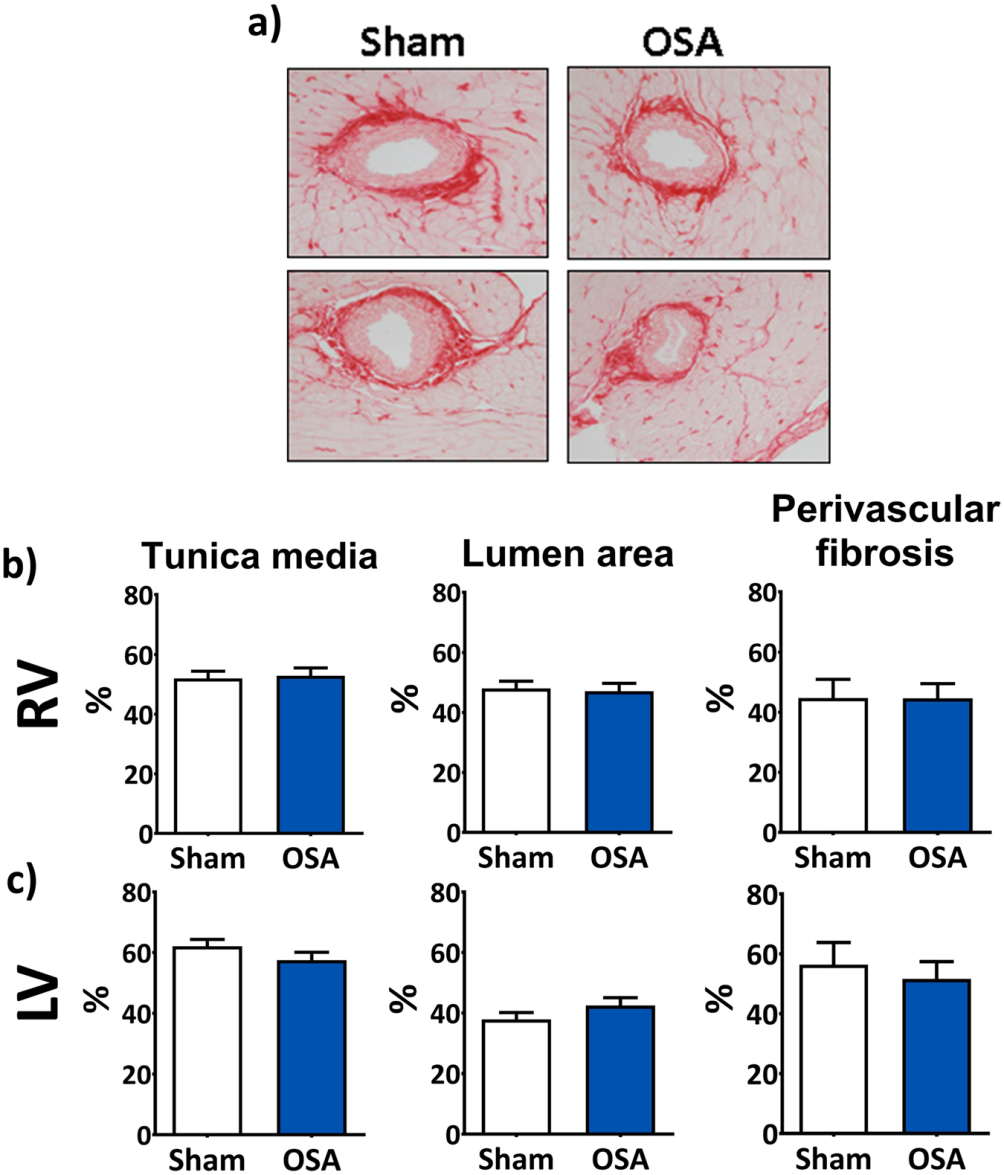
Intramyocardial vessels remodelling. Representative picrosirius-stained pictures of intramyocardial arteries of Sham and OSA rats (**a**). Percentage (%) of tunica media area relative to vessel size, percentage of lumen area relative to vessel size, and percentage of perivascular fibrosis relative to vessel size in vessels from right ventricle (RV, **b**) and from left ventricle (LV, **c**). Data are expressed as a percentage.

To assess the morphological remodelling of muscular arteries, we analysed the lumen, tunica media and perivascular areas of both left and right ventricle mid-ventricular intramyocardial arteries (Fig. 3a). We did not find any changes in the relative size of the tunica media nor of the lumen, nor in the amount of perivascular fibrosis in muscular arteries from OSA rats compared with sham. The lack of changes was consistent across the right (Fig. 3b) and the left ventricle (Fig. 3c).

### Mechanisms of OSA-induced vascular remodelling

**Figure 4:**
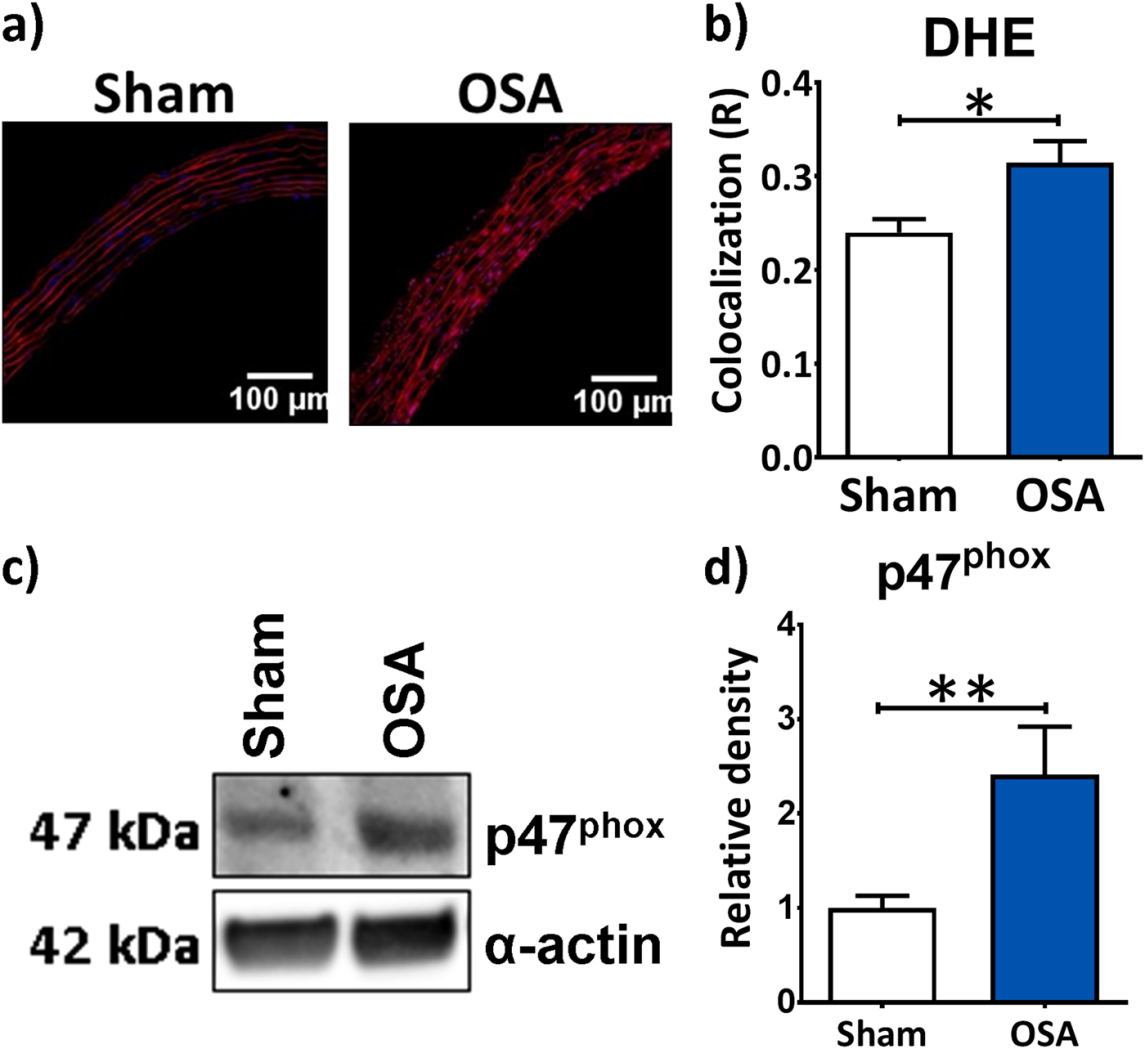
OSA-induced aortic oxidative stress. Representative fluorescence images of aortic OCT sections of Sham and OSA rats (**a**). Quantification of colocalization index (DHE (R)) of dihidroethidium (DHE, red fluorescent signal) and nucleus (DAPI, blue) in frozen aortic sections from Sham (n = 12, white columns) and OSA (n = 15, blue columns) (**b**). Representative immunoblots of the NADPH oxidase subunit p47^phox^ and α-actin (**c**). Column graph of the p47^phox^ (**d**), relative to α-actin band densities from aorta homogenates of Sham (n = 7, white columns) and OSA rats (n = 5, blue columns). Data are expressed as mean ± SEM, *p < 0.05, **p < 0.01. A p47^phox^ western blot membrane is shown in Supplementary Figure 2.

Oxidative stress and both nitric oxide and renin-angiotensin pathways were explored as potential contributors to vascular remodelling in OSA. Superoxide production in the aortic tunica media was estimated with dihydroethidium (DHE) fluorescence (Fig. 4a). Analyses demonstrated increased DHE fluorescence in the OSA compared to the Sham group (Fig. 4b). Protein levels of the NADPH oxidase subunit p47-phox were higher in aortas from OSA compared to Sham rats (Fig. 4c,d).

**Figure 5:**
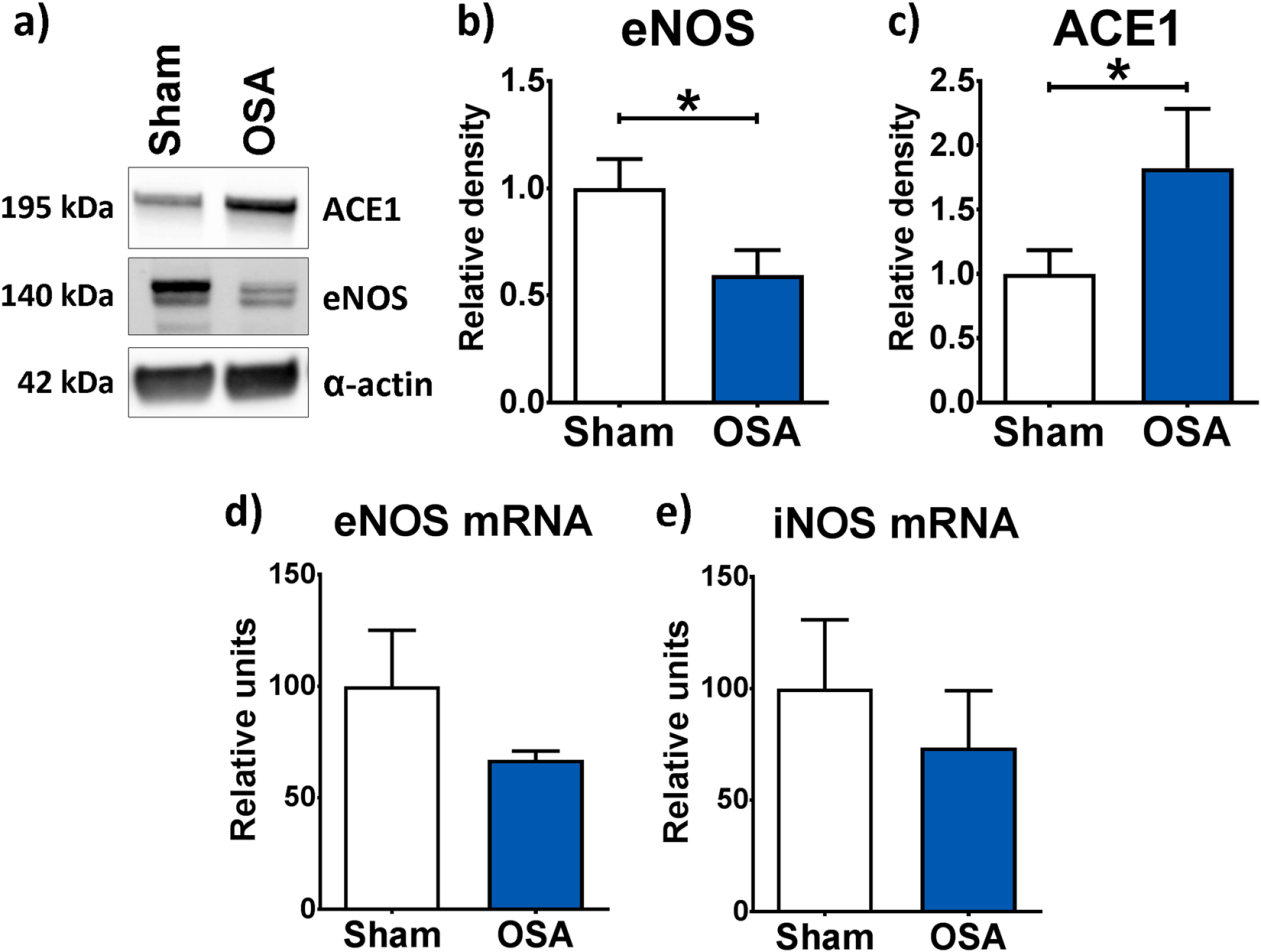
Mechanisms of OSA-induced aortic remodelling. Representative immunoblots of angiotensin converting enzyme-1 (ACE1), endothelial oxide nitric synthase (eNOS), and α-actin (**a**). Mean ± SEM protein levels of eNOS (**b**) and ACE1 (**c**) relative to α-actin from aorta homogenates of Sham (n = 7, white columns) and OSA rats (n = 5, blue columns). A eNOS and ACE1 western blot membranes are shown in Supplementary Figure 2. Mean ± SEM mRNA levels of eNOS (**d**) and iNOS (**e**) relative to *Ppib* from aorta homogenates of Sham (n = 7, white columns) and OSA rats (n = 6, blue columns). *p < 0.05.

Enzymes involved in nitric oxide were also quantified. The OSA group showed decreased eNOS protein (Fig. 5a,b) levels compared to the Sham group, while reduction in eNOS mRNA expression was not significant, suggesting post-transcriptional regulation mechanisms (Fig. 5d). Messenger RNA levels of iNOS were similar between groups (Fig. 5e). Elevated ACE1 protein levels in OSA rats suggested renin-angiotensin-aldosterone system enhancement (Fig. 5a,c).

### Mesenchymal Stem Cell infusions blunt OSA-induced vascular changes

**Figure 6:**
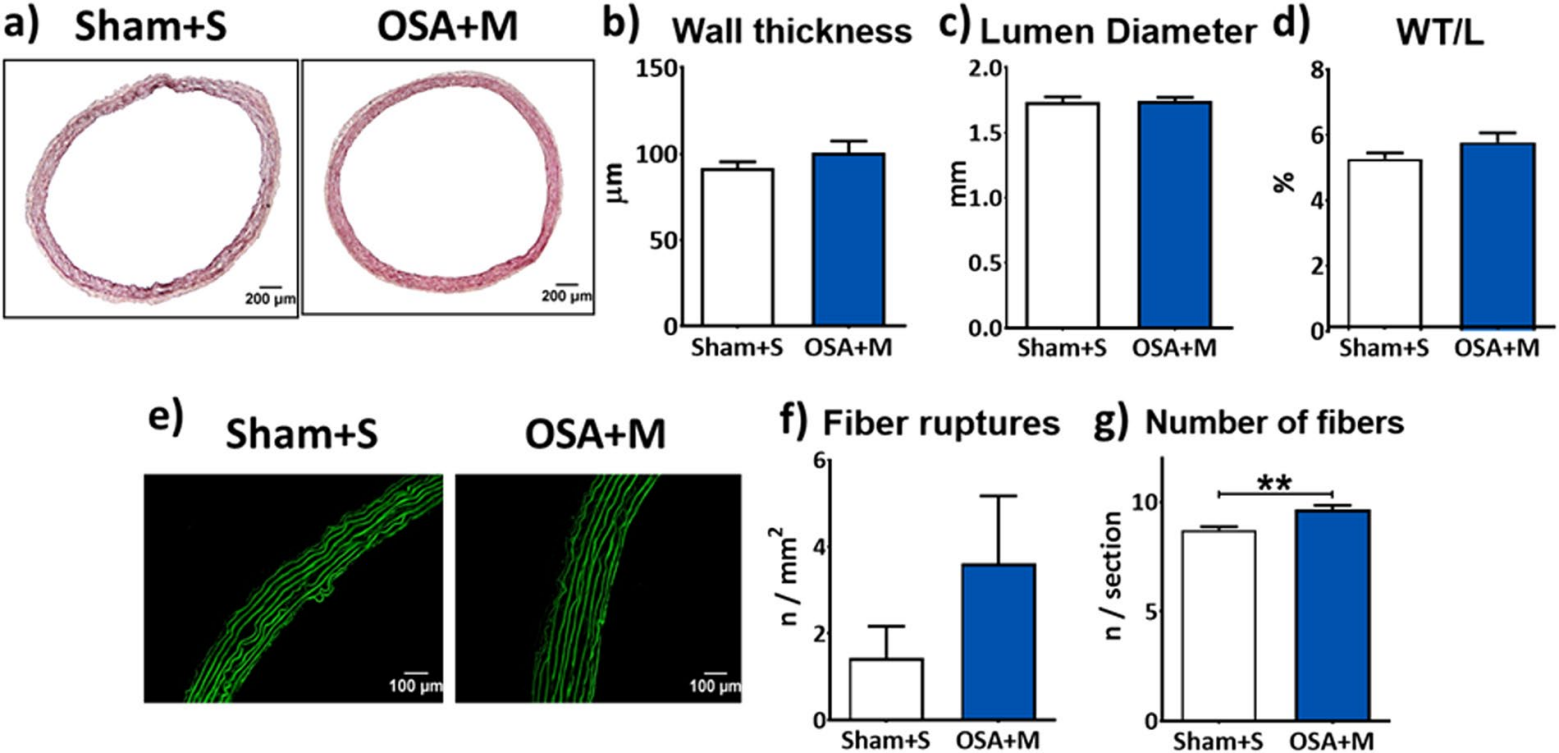
Mesenchymal stem cells infusion normalizes OSA-induced morphological vascular remodelling. Representative haematoxylin-eosin stained aortic sections from Sham rats injected with saline vehicle (Sham + S) and OSA rats injected with mesenchymal stem cells (OSA + M) (**a**). Aortic wall thickness (µm, **b**), lumen diameter (mm, **c**), percentage of wall to lumen ratio (% WT/L, **d**) from sham rats injected with saline vehicle (Sham + S, n = 6, white columns) and from OSA rats injected with mesenchymal stem cells (OSA + M, n = 5, blue columns). Representative elastin autofluorescence images from aorta wall from Sham + S and OSA + M rats (**e**). Number of fiber ruptures indexed for area (mm^2^) in Sham + S (n = 8, white columns) and OSA + M (n = 7, blue columns) groups (**f**). Number of elastic fibers relative to each aorta section in the same samples as in F (**g**).

To analyse the effect of mesenchymal stem cell (MSC) infusions in OSA-induced aortic structural remodelling, morphological parameters were studied in aortic sections from Sham rats infused with saline vehicle (Sham + S) and rats subjected to the airway obstructions and MSC infusions (OSA + M). MSC infusion reverted most OSA-induced aortic structural changes. No differences in WT (Fig. 6a,b), lumen diameter (Fig. 6a,c), and the WT/L (Fig. 6a,d) were observed between groups. Also, the number of elastic fiber ruptures per area was not statistically different between aortas from Sham + S and OSA + M rats (Fig. 6e,f). Nevertheless, the number of elastic fibers per section remained elevated in OSA + M group (Fig. 6e,g).

**Figure 7:**
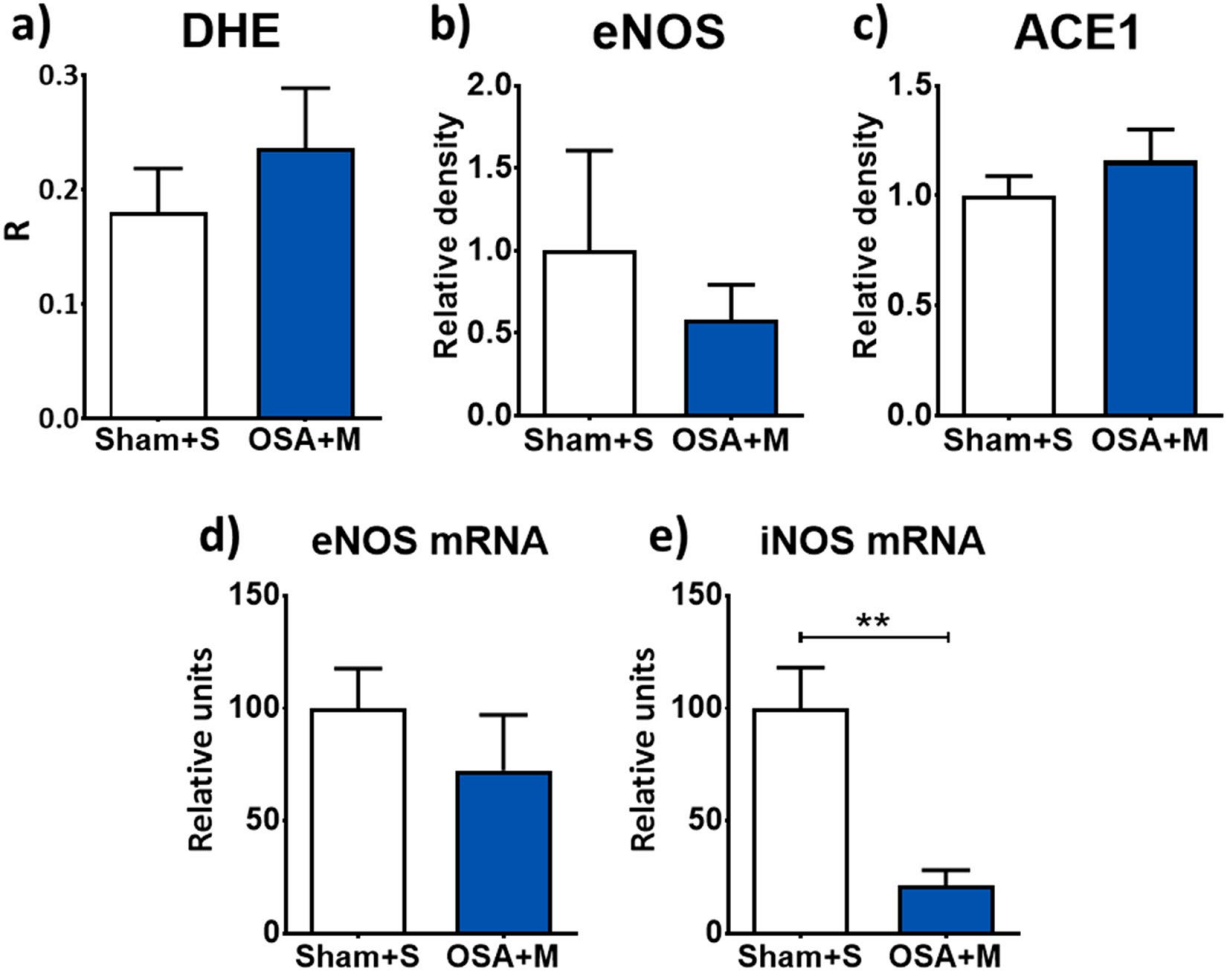
Mesenchymal stem cells infusion normalizes mechanisms of OSA-induced vascular remodelling. Quantification of the colocalization index of dihidroethidium and nucleus (DHE [R]) in OCT aortic frozen sections in Sham rats infused with saline vehicle (n = 7, Sham + S) and OSA rats injected with mesenchymal stem cells (n = 8, OSA + M) (**a**). Mean ± SEM protein levels of eNOS (**b**) and of ACE1 relative to α-actin in Sham + S (n = 8) and OSA + M (n = 8) rats (**c**). Mean ± SEM mRNA levels of eNOS (**d**) and iNOS (**e**) relative to *Ppib* from aorta homogenates of Sham + S (n = 8, white columns) and OSA + M rats (n = 6, blue columns). **p < 0.01.

Increased aortic superoxide generation by OSA was prevented by the administration of MSC, yielding similar levels of intranuclear DHE fluorescence in both Sham + S and OSA + M rats (Fig. 7a). Similarly, eNOS protein (Fig. 7b) and mRNA levels (Fig. 7d) were similar between groups after MSC administration. Conversely, MSC infusion dramatically decreased iNOS mRNA expression (Fig. 7e). ACE1 protein levels were comparable in Sham + S and OSA + M rats (Fig. 7c).

## Discussion

This study demonstrates for the first time that chronic, non-invasive repetitive airway obstructions mimicking OSA promote remarkable structural changes of the descending thoracic aorta as well as an increase in reactive oxygen species (ROS), endothelial dysfunction and renin-angiotensin system (RAS) markers. Our results also indicate that MSC infusion blunts OSA-related vascular changes.

We demonstrate that OSA-induced aortic structural remodelling is characterized by lumen dilatation and hypertrophy of the vessel wall, an increased number of elastic fibers, and a higher number of fiber ruptures. In accordance with our results, exposure to IH has been shown to induce an expansive aortic remodelling with intima-media thickening (IMT) in 14-day^16^ and 6-week^17^ mice models. In these models, increased elastic fiber disorganization and fragmentation accompanied wall thickening. Also, an increase of tunica media thickness along with elastic fibers ruptures and disorganization have been demonstrated in a canine invasive OSA model of 12 weeks of duration^18^. Perturbations in the continuity of elastic lamina with disruption of the elastic fibers are apparent during early stages of atherogenesis in apoE knockout (apoE−/−) mice and may act as a potential substrate for subsequent development of aortic lesions^19^.

The realistic animal model used in this work mimics recurrent obstructive apneas observed in OSA patients. The animals were subjected to both intermittent hypoxia-reoxygenation events, like in IH models, and negative intrathoracic pressure swings associated with strenuous breathing efforts. We have previously shown that this model results in an arterial oxygen saturation (SaO_2_) fall to ≈80% associated with intermittent hypercapnia^9^. We hypothesize that the OSA-related increased lumen diameter in our work may be linked to the intrathoracic pressure swings observed in OSA. Those pressure swings cause shear and wall stress in intrathoracic blood vessels^20^, altering its mechanical properties and ultimately resulting in aortic dilatation^21^. In addition to the increased intrathoracic pressure swings, the airway obstructions in our OSA model mimicked the intermittent hypercapnia events as experienced in patients. In this context, it has been recently suggested that the combination of hypoxia with hypercapnia in apoE and low density lipoprotein receptor Ldlr-deficient mice might accelerate the progression of atherosclerosis in the aorta and in the pulmonary artery^22^.

The lack of neither a collagen increase nor shift in collagen I and III percentage in the aortas of our 21-day OSA model, together with the results from the literature, suggests that collagen deposition is a late event of vascular remodelling triggered by repeated hypoxia episodes. No changes in collagen content were reported after 14 days of IH exposure^16^, while an extension of the IH time exposure up to 6 weeks^17,23^ or an invasive OSA model up to 12 weeks^18^ led to an elevated accumulation of collagen fibers in the aortas.

In our study we did not find changes in tunica media thickness or perivascular fibrosis in muscular intramyocardial vessels. Again, results from the 6 weeks exposure to IH reveal higher perivascular fibrosis in intramyocardial vessels^17,23^ indicating that collagen deposition in those vessels could be related to a longer IH exposure.

In human studies, patients with OSA display signs of vascular remodelling, including increased carotid IMT and increased arterial stiffness compared to control subjects^2,24^. Nevertheless, the association between OSA and the ascending aorta diameter in humans remains controversial. Most studies reported a positive association between the aorta diameter and parameters of OSA severity^21,25,26^ although conflicting data has also been published^27,28^, likely because of heterogeneity in study designs and cohort composition.

Intermittent hypoxia in OSA is associated with increased levels of ROS and elevated production of soluble adhesion molecules and pro-inflammatory cytokines^29^, and is thought to be one of the main components linking OSA to cardiovascular disease and atherosclerosis^30^.

Our results showed enhanced aortic superoxide anion generation, one of the most important ROS species within the vasculature, in OSA rats compared to Sham rats. The NADPH oxidase is a major source of ROS^31^. We observed an increase of the aortic protein levels of NADPH oxidase subunit p47^phox^ in OSA rats, in agreement with an elevated superoxide production. Our data is consistent with the increased levels of oxidative stress markers^32,33^, lipid peroxidation, protein carbonylation and DNA oxidation and an impaired antioxidant capacity in OSA patients^34^.

Our data showed decreased aortic eNOS protein levels and no changes in iNOS mRNA levels in OSA compared to Sham rats, suggesting a reduced NO bioavailability. These results are in agreement with Jelic *et al*. who reported reduced baseline expression of eNOS and phosphorylated eNOS in endothelial cells harvested from patients with OSA compared with control subjects. Consistently, flow-mediated dilation was found to be significantly decreased in these patients^35^.

We showed elevated aortic ACE1 protein levels in OSA rats. Angiotensin II (Ang II), generated by the conversion of Angiotensin I through ACE1, has multiple local and systemic effects that finally lead to vasoconstriction, inflammation and smooth muscle cell proliferation, thus contributing to the process of atherosclerosis^36^. RAS activation is a well-known signalling pathway implicated in blood pressure control. It has been proposed that OSA could contribute to hypertension, observed in approximately 50% of OSA patients, via RAS activation^37,38^. In an experimental study, 35 days of consecutive recurrent hypoxia (30 s, 7 h/day) promoted 8–13 mmHg increase of mean arterial blood pressure in rats, which was blunted with the AT1 receptor blocker losartan^39^. NADPH oxidase is a downstream effector of Ang II^40,41^, suggesting that the increased ACE1 levels may impinge on the NADPH oxidase subunits levels and ROS markers discussed previously. Our findings are also in accordance to recent works showing that chronic IH exposures lead to the recruitment of CD36(+)high macrophages to the aortic wall promoting atherogenesis^42,43^.

Taking into consideration that only 30–60% of patients are adherent to continuous positive airway pressure therapy^44^, future new therapeutic approaches in order to prevent vascular damage in OSA patients are required. Studies have demonstrated anti-inflammatory properties of MSC by regulation of the proliferation and function of different immune cell types^45,46^. In terms of OSA, we observed an early release of MSC into peripheral blood in response to recurrent obstructions in rats^15^. In addition, we found that MSC triggered an anti-inflammatory response in the same OSA animal model^12,13^. Although the available data on the role of MSC is still very scarce, it has been suggested that the anti-inflammatory properties of MSC could counterbalance the OSA-induced adverse structural remodelling. In this context, after MSC infusion we observed a regression of OSA-induced morphological aortic changes, superoxide anion production, eNOS and ACE1 protein levels imbalance and a reduction of iNOS mRNA levels.

In the present work, similar aortic size was found in OSA rats treated with MSC compared to the Sham group. In agreement with our results, in a rat model of abdominal aortic aneurism (AA), an endovascular xenograft of bone marrow MSCs (BM-MSCs) decreased AA diameter expansion^47^. Additionally, in apoE−/− mice administrations of BM-MSCs attenuated the development of Ang II-induced AA^48,49^. Furthermore, our data shows that MSC infusion was able to counterbalance the disruption of elastic fibers associated with OSA, allowing the preservation of the aortic wall structure. Similarly, data from Hashizume *et al*.^49^ indicate that implantation of MSCs contributes to the attenuation of experimental AA growth through elastin preservation in the aortic wall associated with down regulation of metalloproteinases and inflammatory cytokines *in vivo*.

Therefore, MSC may serve as a future therapeutic strategy for OSA patients. However, further experiments are needed to confirm the effects of multiple intravenous administrations of MSC as well as the detailed mechanisms by which MSC exert such a therapeutic action, in particular whether MSC conditioned medium or MSC-extracted exosomes could play a similarly effective role^50,51^.

In conclusion, in this clinically relevant model, chronic OSA causes outward hypertrophy of descending thoracic aorta and elastin disorganization by a mechanism that may involve increased oxidative stress, ACE1 up-regulation and eNOS down-regulation. MSC infusion reverses aortic structural changes and attenuates superoxide anion production and ACE1 and eNOS changes. Our results provide valuable insights into the biological mechanisms that promote vascular damage in OSA and suggest that MSC could counterbalance oxidative stress, RAS up regulation and endothelial dysfunction in OSA, thus normalizing OSA-induced structural vascular remodelling. Further research on the mechanisms underlying OSA-induced aortic remodelling would be of interest.

## Methods

### Experimental sleep apnea model

The experimental protocols were approved by the Animal Research Ethics Committee of the University of Barcelona and by the Generalitat de Catalunya with the protocol number 5628. It also conformed to European Community Directive (2010/63/UE) and Spanish guidelines (RD 53/2013) for the use of experimental animals.

A chronic model of OSA previously validated by our group was used^9^. In the first experiment, thirty Sprague-Dawley male rats (250–300 g) were randomized into 2 groups: OSA rats (n = 16) were subjected to 15-second obstructions (60/hour, 6 hours/day, 21 days) in the custom-made setup, and Sham rats (n = 14) were placed in the setup without air obstructions. Rats in both groups were progressively adapted to the experimental setting by increasing the time within the custom-made setup at a rate of 1 hour each day, up to 6 hours at the end of the first week.

### Mesenchymal stem cells infusion

To evaluate the effect of MSC, a subsequent set of 16 Sprague-Dawley male rats (250–300 g) were randomized into 2 groups: 8 rats undergoing the OSA protocol for 21 days were infused with MSC (OSA + M). Eight Sham rats receiving saline vehicle (Sham + S) were used as controls in this experiment.

MSC from Lewis rat marrow stromal cells were kindly provided by the Tulane Center for Gene Therapy (New Orleans, LA, USA). The cells were cultured and 5 × 10^6^ cells were injected the first day of apneas application and every 4 days thereafter, as explained in a previous work^12^.

All rats were carefully inspected daily during the OSA experiment and none showed any signs of stress or MSC-injection related side effects.

### Euthanasia and sample collection

After 21 days, rats were anesthetized with intraperitoneal urethane 10% (1 g/kg) and sacrificed by exsanguination through carotid artery catheterization. Descending thoracic aorta was rapidly immersed in OCT and frozen for histological measurements and RNA extraction or snap-frozen in liquid nitrogen for western blot analyses.

### Morphologic analysis of aorta

Haematoxylin-eosin staining was performed in 4-µm frozen OCT-sections of descending thoracic aorta. Microphotographs of full aorta sections were obtained (4X) with an Olympus BX41TF light microscopy, a DP73 camera (Olympus Corporation, Japan) and image analyser Olympus cellSens Standard 1.16 (Olympus Corporation). All the histological measurements were quantified with the ImageJ software (NIH, Maryland, USA)^52^ by an investigator blinded to group assignment.

The internal perimeter (lumen) and external perimeter (external elastic membrane) of the vessel were manually drawn and their length measured. It was assumed that cross-sectional area was circular in *in vivo* conditions and that both perimeter values corresponded to external and internal circumference length (C). The internal (D_i_) and external diameters (D_e_) were determined from the equation D = C/π. Aorta wall thickness (WT) was then calculated as WT = (D_e_ − D_i_)/2. The WT to lumen ratio (WT/L, in %) was calculated to estimate wall thickness relative to lumen.

### Elastic fiber number and disruption

Frozen aortic OCT-sections (10 μm-thick) were used to analyse elastic laminae by means of its auto fluorescence, as described in de Thomaz^53^. Briefly, a set of 3–4 images (40X) of elastin green auto fluorescence (excitation 351–364 nm and emission 400–500 nm) were used to assess aortic elastic fiber architecture, both in the form of discontinuities and number of fibers. Discontinuity was defined as a complete rupture of an elastic fiber, with boundaries at both sides clearly visible. Number of discontinuities were quantified and indexed for area (mm^2^). Elastic fiber number relative to each aorta section area was also quantified.

### Aorta RNA isolation and real time PCR analysis

The remaining of the frozen aortic OCT-sections were incubated with 0.5 mL of Trizol until the OCT was dissolved. The tissue was then transferred to a new tube with 0.5 mL of Trizol to proceed with tissue disruption followed with silica column purification with the *mir*Vana miRNA Isolation Kit protocol (Invitrogen). Total RNA (0.15 μg) was retrotranscripted with the High-Capacity cDNA Reverse Transcription Kit (Thermo Fisher). eNOS and iNOS mRNA levels were measured with Real time PCR with the enzyme SYBR Select Master Mix (4472908, Thermo Fisher). Real time PCR results were normalized to the cyclophilin B (*Ppib*) housekeeping gene, and presented with the 2^−ΔΔCt^ method. Real time PCR details are listed in Table 1 below.

**Table 1:** Real time PCR details of the three mRNA analyzed, NCBI reference is the sequence used for the primers design, sequences of both the forward and reverse primers and their annealing temperature (aT).

mRNA	NCBI reference	Forward primer	Reverse Primer	aT
eNOS	NM_021838	GGATCCAGTGGGGGAAACTG	TGGCTGAACGAAGATTGCCT	60
iNOS	NM_012611.3	GGCAGGATTCAGTGGTCCAA	GAACATTTCTGATGCAGTGCTACA	60
PPIB	NM_022536.2	AGCGCAATATGAAGGTGCTCT	CTTATCGTTGGCCACGGAGG	60

### Aorta protein isolation and western blot analysis

Frozen aortas were submerged in 0.5 mL of ice-cold RIPA Lysis buffer (89901, Thermo-Fisher Scientific, MA, USA) and Halt Protease Inhibitor (1862209, Thermo-Fisher Scientific), and homogenized with an Omni TH homogenizer (Omni International Inc.). After 1 hour of rotation at 4 °C, samples were centrifuged at 10.000 g at 4 °C for 30 minutes. The upper phase was collected and the total protein concentration was quantified with the Pierce^TM^ BCA protein Assay method (23227, Thermo-Fisher Scientific, MA, USA) relative to a BSA standard curve. Equal amounts of protein (25 μg) from each aorta were resolved by standard Novex gels methodology (Thermo-Fisher Scientific, MA, USA) as described in Ramos *et al*.^12^.

To analyse protein levels, the following primary antibodies were used: rabbit polyclonal anti-Angiotensin Converting Enzyme 1 (ACE-1, F949) (BS3485, Bioworld Technology, MN, USA), mouse anti-eNOS/NOS Type III (610297, BD Biosciences) and rabbit polyclonal anti-p47-phox (H-195) (sc-14015, Santa Cruz Biotechnology Inc.). Protein loading normalization was performed against α-actin housekeeping protein using the mouse monoclonal antibody anti-Smooth muscle actin (clone 1A4) (M0851, Dako Diagnostics SA., Denmark). Molecular weight of the measured bands is shown in a separate set of rats in Supplementary Figure 1.

### Aortic fibrosis quantification

Four micron-thick aortic OCT-sections were stained with Picrosirius-red to determine collagen deposition. Four random pictures (40X) from each aortic section were obtained and the collagen fraction of the tunica media was assessed with a semiautomatic colour-threshold detection method. Collagen content was calculated as a percentage of the total area. Adventitial fibrosis was excluded from the analysis.

The same sections were analysed under polarized light with a Leica DMRB microscope, Leica DFC450 camera and the Leica software LAS V4.3 in the Confocal Microscopy Core facility (Universitat de Barcelona). Four to five random pictures (20X) from each aorta were obtained and collagen I was quantified with the hue definition 0–10 and 233–255 that identifies red staining. Collagen III was quantified with the hue definition 15–138 that identifies green staining. The colour definitions were adapted to our images as described previously^54^. All collagen quantifications were performed blinded to group assignment, using Image J software for Windows^52^.

### Superoxide detection in aortic sections

The oxidative fluorescent dye dihydroethidium (DHE) was used to evaluate *in situ* production of superoxide anion in frozen unfixed aortic OCT-sections (10 μm-thick). Briefly, sections were equilibrated for 30 min at 37 °C in a HEPES buffer solution (pH 7.4) and incubated for 30 min in a light-protected and humidified chamber at 37 °C with 5-μM DHE solution (D7008, Sigma-Aldrich). After DHE was washed, coverslips were mounted using ProLong Gold antifade reagent with DAPI (P36931, Thermo-Fisher Scientific). Images were recorded at different excitation wavelengths using the same settings in all samples: 350 nm (blue for DAPI) and 590 nm (red for 2-hydroxyethidium). In order to measure the amount of colocalization between the two dyes, Pearson’s correlation coefficient (R) was calculated using ImageJ 1.48 v software (colocalization analysis plugin) in four areas per each aortic ring.

### Morphologic analysis of intramyocardial arteries

Mid-ventricular 4-µm sections were stained with Picrosirius-red. Random pictures of left ventricle (LV) and right ventricle (RV) were taken from each sample (10X). For each sample, 2 to 4 intramyocardial coronary arteries were localized in both LV and RV. Three different areas were quantified for each vessel: perivascular area (A), limited by outer tunica adventitia; external area (Ae), limited by outer tunica media; and internal area (Ai), limited by internal elastic lamina. All measures are given as a percentage relative to the vessel area. The percentage of lumen area was quantified as [(Ai/Ae) × 100], the percentage of tunica media as [(Ae − Ai)/Ae × 100], and the percentage of perivascular fibrosis as [(A − Ae)/Ae) × 100)].

### Statistical analyses

Data are expressed as mean ± standard error of the mean (SEM) or as percentages (%). Data adjustment to normality was checked with a residual Q-Q plot. Comparisons between 2 groups were carried out with the non-paired Student’s t-test or the non-parametric U Mann-Whitney test when appropriate. *P* < 0.05 was considered to be statistically significant. The statistical analysis was carried out using the Prism 5 software (GraphPad Software, San Diego, CA, USA).

## Supplementary information

supplementary information

## Data Availability

The datasets used and/or analysed during the current study are available from the corresponding author on reasonable request.
