# Supplementary material for: Aortic remodelling induced by obstructive apneas is normalized with mesenchymal stem cells infusion
Source: Sci Rep. 2019 Aug 7;9:11443. doi: 10.1038/s41598-019-47813-1 (PMC6685984; doi:10.1038/s41598-019-47813-1)
Supplement: Supplementary file 1 — supplementary information [file 41598_2019_47813_MOESM1_ESM.pdf]

Aortic remodeling induced by obstructive apneas is normalized with mesenchymal stem cells infusion

Cira Rubies PhD, Ana-Paula Dantas PhD, Montserrat Batlle PhD , Marta Torres PhD, Ramon Farre PhD, Gemma Sangüesa PhD, Josep M Montserrat MD, PhD, Lluis Mont MD, PhD, Isaac Almendros PhD, Eduard Guasch MD, PhD

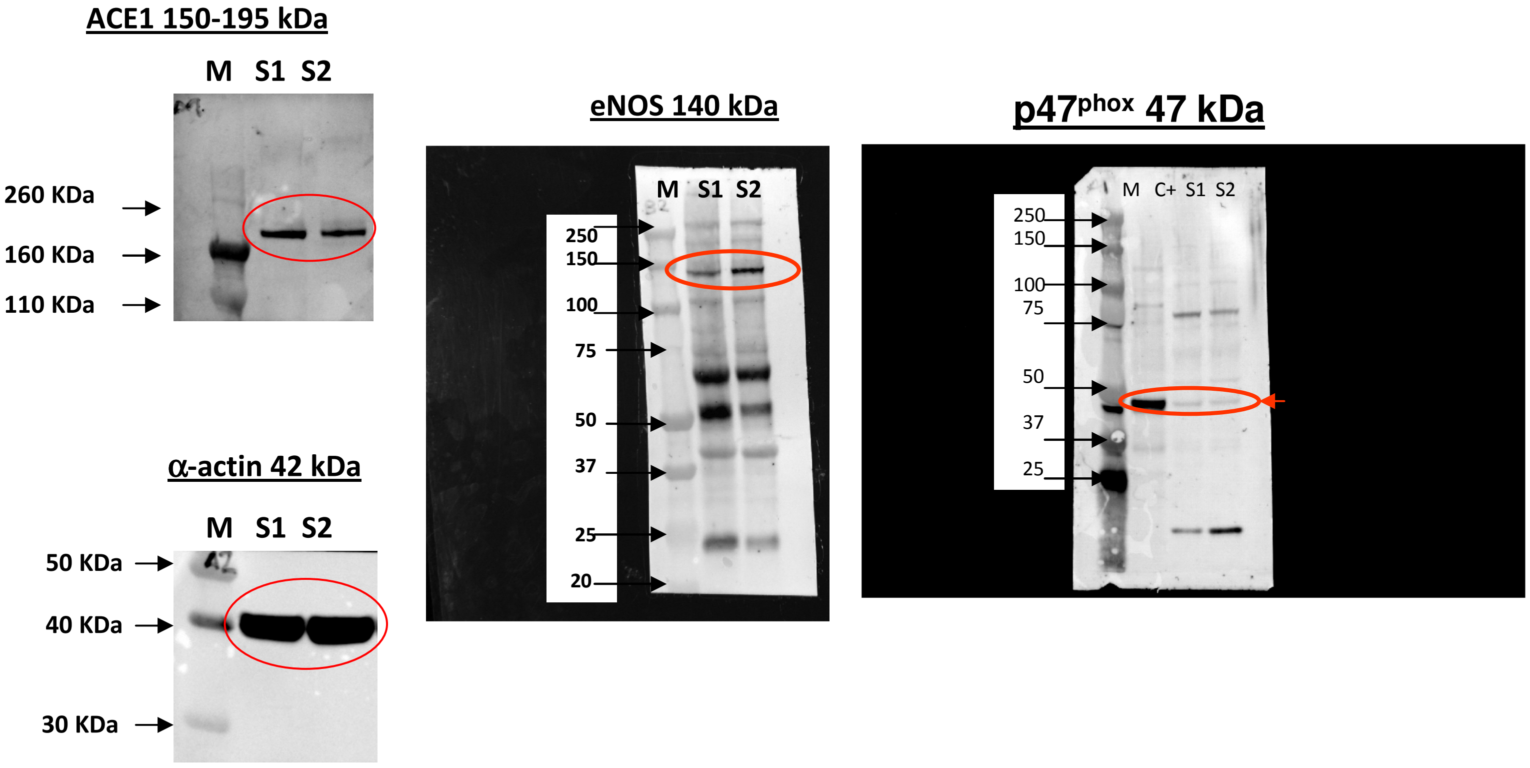

Supplementary figure 1: Molecular weight marker (M) and aorta protein homogenate from two naïve rats (not included in other analyses). Western-blots for ACE1, eNOS, α-actin and p47phox.

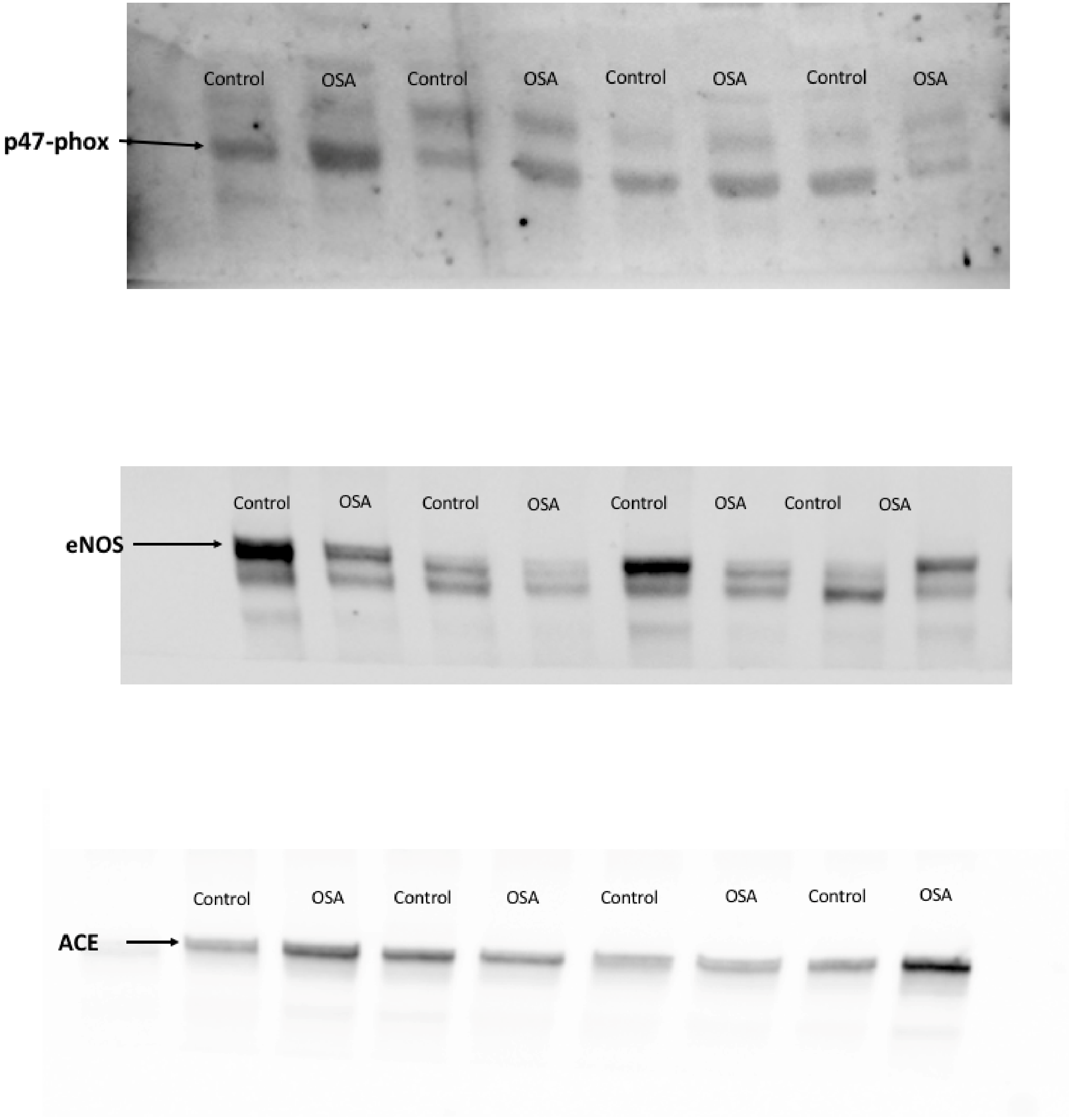

Supplementary figure 2: Western-blots for p47phox (quantified in figure 4d), eNOS and ACE1 (quantified in figure 5b and 5c respectively).
